# Editorial: Targeting Leukocyte Trafficking: Insights and Future Directions

**DOI:** 10.3389/fimmu.2021.777002

**Published:** 2021-10-05

**Authors:** Ronen Sumagin, Sian M. Henson, Vicky L. Morrison, Helen M. McGettrick

**Affiliations:** ^1^ Department of Pathology, Feinberg School of Medicine, Northwestern University, Chicago, IL, United States; ^2^ Translational Medicine and Therapeutics, William Harvey Research Institute, Barts and The London School of Medicine and Dentistry, Queen Mary University London, London, United Kingdom; ^3^ Institute of Infection, Immunity and Inflammation, University of Glasgow, Glasgow, United Kingdom; ^4^ Institute of Inflammation and Ageing, University of Birmingham, Birmingham, United Kingdom

**Keywords:** adhesion, ageing, chronic inflammatory diseases, Integrins, leukocyte, leukocyte recruitment, migration, T-cells

The ability of leukocytes to exit the vasculature (blood, lymphatic) and enter tissue (lymphoid, peripheral) is critical for the health and wellbeing of an organism. These processes form a tightly regulated cascade of sequential steps, each of which educates the migrating leukocyte. Whilst some believe that our knowledge of the leukocyte adhesion/migration cascade is complete, advances in imaging technologies and genetically modified animals and cell-lines, amongst others, are still revealing new insights to the original cascade described in 1995. For instance, unbiased omics approaches coupled with interactome mapping are expanding our appreciation of the cytoskeletal proteins, signalling events, and phosphorylation patterns coupled to integrin-mediated migration and effector function in leukocytes. Our current understanding of downstream inside-out and outside-in signalling through β_2_-integrins in neutrophils and the consequence of phosphoinositide-3-kinase (PI3K) signalling in T-cell trafficking are elegantly reviewed by Bouti, et al. and Johansen, et al., respectively. Moreover, molecules traditionally considered to elicit effector functions are now being directly linked to the regulation of leukocyte migration. Here, myeloperoxidase (MPO) has been reported to negatively regulate neutrophil infiltration during inflammation and its absence significantly enhanced the migration capacity of neutrophils (Rehring, et al.).

Vascular endothelial cells are key gatekeepers of the tissue: with blood endothelial cells (BEC) facilitating leukocyte ingress and lymphatic endothelial cells (LEC) supporting their tissue egress. Renewed interest in glycan-binding proteins has revealed that specific expression patterns of galectin family members on BEC from different tissues or in response to different stimuli (e.g., reviewed by Lightfoot, et al.). Although there is still much to learn about galectins, such differences are likely to have a big impact on leukocyte-BEC interactions and thus leukocyte entry into inflamed tissues. Focusing on tissue egress Tadayon et al., have describe a dual role for Clever-1: (i) acting as a key adhesion molecule to support dendritic cell (DC) migration across LEC; whilst also (ii) negatively regulating the inflammatory phenotype of LEC (Tadayon, et al.). Consequently, LEC Clever-1 educates migrating DC, imparting an immunosuppressive signal that regulates the magnitude of immune response created in the draining lymph nodes.

Leukocyte migration is unequivocally linked to their ability to mediate their effector functions – whether this is acting as first responders to invading pathogens and/or tissue injury in peripheral tissues; mounting highly specific antigen driven detection systems in lymphoid tissues; or regulating the degree and duration of such responses. Deficiencies in genes responsible for actin regulation, which is crucial for cellular movement, are associated with primary immunodeficiencies, including megakaryoblastic leukaemia (MKL1) (Sprenkeler, et al.). MKL1 deficiency is characterised by severe defects in neutrophil adhesion and migration, as well as the potential for myeloid cells to spread and form filipodia to sense chemotactic and haptotactic gradients (reviewed by Sprenkeler, et al.). Additionally, changes in adhesion molecule expression can provide novel insights into microbial responses, for example SARS-CoV-2 infection significantly reduced the expression of the gut-homing integrin, α_4_β_7_, on circulating blood lymphocytes, indicative of priming in the GALT and potentially the preferential recruitment of these cells from blood into peripheral tissues (Müller, et al.).

Conversely, the inappropriate accumulation and activation of leukocytes underpins pathology and tissue damage in numerous immune-mediated inflammatory diseases, such as rheumatoid arthritis (Wright, et al.); chronic obstructive pulmonary disease (Belchamber, et al.); and inflammatory bowel disease (Wiendl, et al.). RNAseq analysis has revealed that rheumatoid synovial fluid fundamentally alters the migratory potential of healthy neutrophils, contributing to their accumulation, retention and the maintenance of an activated phenotype in the rheumatoid joint (Wright, et al.). Similarly, dyslipidaemia associated with atherosclerosis, in particular low levels of ApoA1, were correlated with elevated expression of integrin and chemokine receptors on circulating monocytes (Patel, et al.), thus supporting aberrant monocyte extravasation and potentially driving plaque progression.

Our ability to target leukocyte migration offers the opportunity to influence leukocyte trafficking profiles based on the underlying defect. For example, promoting recruitment in individuals where responses have diminished, e.g., in the primary immunodeficiencies (Sprenkeler, et al.), the elderly (Nathan, et al.) or in cancer (Bonilha, et al.). Additionally, one could envisage clinically taking advantage of the diurnal rhythmicity of leukocyte trafficking to maximise these responses (Nathan, et al.). Alternatively, treatment options might include limiting the trafficking of pathogenic effector leukocytes at sites of chronic inflammation, combined with agents that promote the migration of regulatory leukocytes to trigger resolution of inflammation and tissue repair. These concepts are elegantly highlighted by Bonilha et al., where antagonising junctional adhesion molecule- A (JAM-A) may control leukocyte trafficking in preclinical models of IMIDs, whilst the same treatment appears to enhance immune responses against cancerous cells in tumors (Bonilha, et al.).

Caution is required when designing any therapy that targets leukocyte adhesion and migration: Many of the molecules involved in these processes are ubiquitously expressed and utilised by all leukocyte’s subtypes in their movement around the body. One of the major therapeutic success stories is vedolizumab, the anti-α_4_β_7_-integrin antibody, used in IBD, which takes advantage of the unique adhesion molecule profile in the gut to specifically prevent the trafficking of gut-homing T-cells into the mucosal tissue (reviewed by Wiendl, et al.). Opportunistic infection and poor vaccine responses are common side-effects to many of the biologics in development or that have been developed, which is not surprising when considering the clinical attributes of primary immunodeficiencies. Moreover, attempts to target leukocyte accumulation by modulating their recruitment with chemokine receptor inhibitors has been unsuccessful in a number of contexts, including COPD (Belchamber, et al.), due to promiscuity and redundancy within this family. Further directions should focus on interactions that are unique to “disease” settings to minimise off-target effects, but also attempt to identify shared pathways hijacked across multiple conditions that might benefit from early, preclinical interventions.

The Frontiers Research Topic “*Targeting Leukocyte Trafficking: Insights and Future Directions*” includes a combination of reviews and original papers highlighting advances in our fundamental understanding of the molecular processes regulating leukocyte trafficking in health, during a day or over a lifespan, and in response to infection and disease. Each article further explores how this knowledge has and is being translated into novel pharmacological tools, providing current and future perspectives in this arena. The editorial team would like to thank all those who contributed to this collection.

## Author Contributions

All authors listed have made a substantial, direct, and intellectual contribution to the work and approved it for publication.

## Conflict of Interest

The authors declare that the research was conducted in the absence of any commercial or financial relationships that could be construed as a potential conflict of interest.

## Publisher’s Note

All claims expressed in this article are solely those of the authors and do not necessarily represent those of their affiliated organizations, or those of the publisher, the editors and the reviewers. Any product that may be evaluated in this article, or claim that may be made by its manufacturer, is not guaranteed or endorsed by the publisher.

